# Perceived Discrimination and Life Satisfaction of Elderly Chinese People: The Chain Mediating Effects of National Identity and Sense of Community

**DOI:** 10.3389/fpsyg.2018.02572

**Published:** 2018-12-21

**Authors:** Jin Yao, Liping Yang, Xiaoxia Han, Yiying Li

**Affiliations:** ^1^School of Psychology, Nanjing Normal University, Nanjing, China; ^2^School of Education Science, Huaiyin Normal University, Huai’an, China

**Keywords:** perceived discrimination, national identity, sense of community, life satisfaction, Chinese elderly

## Abstract

In China, aging is becoming an increasingly serious issue, and the Chinese government are paying more attention to the life satisfaction of the elderly. Nevertheless, in their daily lives, the elderly are often discriminated against, which may have a negative impact on their life satisfaction. To enable a better understanding of these relationships, we discuss the factors affecting the macro-system (national identity) and micro-system (sense of community) of the elderly. Three hundred and ninety-one elderly people (60–101 years old; 121 males, 270 females) from three communities in the Anhui and Shandong provinces of China participated in our study. Each participant completed the appropriate questionnaires, including: perceived discrimination measure, national identity questionnaire, sense of community questionnaire, and life satisfaction questionnaire. The results of structural equation modeling revealed that perceived discrimination negatively influenced life satisfaction through national identity and community. Perceived discrimination was found to negatively predict national identity, suggesting that perceived discrimination brings a negative influence to national identity within Chinese culture. The relationship between perceived discrimination and life satisfaction was partially mediated by the chain of national identity and sense of community. The size of the total mediation effect was 32.17%. The relationship between perceived discrimination and life satisfaction, when mediated by national identity or sense of community, was not significant. This suggests that the application of the rejection-identification model to the elderly in China may produce different results. The limitations and the implications of our study were considered in discussion.

## Introduction

Since 1999, the rate of aging in China has grown rapidly. 16.1% of the population is over 60 years old in China, which has exceeded 221 million people so far. According to the current speed of population aging in China, it is estimated that by 2020 the elderly population will reach 255 million, and the extent of population aging in China will become more severe in the next 20 years (Zhai et al., 2017). The elderly are a precious resource of the state and society, and improving their quality of life is an important issue. Life satisfaction is the comprehensive psychological index of individual quality of life (Chen and Yue, 2001), and higher life satisfaction is an important aspect of healthy aging (Xie et al., 2014). Therefore, improving the life satisfaction of the elderly is of great importance.

Life satisfaction refers to the individual’s evaluation of his or her quality of life, based on the standard that person has set. It is the cognitive component of subjective well-being (Shin and Johnson, 1978). Past research on the life satisfaction of the elderly mainly focused on demographic variables (Bowling and Browne, 1990), objective factors (Kahn et al., 2003; Gow et al., 2007) and psychological factors (Heaven, 1989; Huebner, 1991; Mellor et al., 2008). Among these, discrimination against the elderly has been an important psychological factor negatively affecting their life satisfaction. Ageism refers to the stereotyping and unfair treatment of old people due to their actual age or perceptions of them (Iversen et al., 2009). Ageism is very common, such as individuals thinking of the elderly as a social burden, and the rejection of the elderly by service industries. This has not disappeared with the development of China’s social civilization, the aging of its population and the advocacy of active aging, but it has become more implicit. It also presents some difficulties for the investigation of ageism from an objective perspective. Therefore, it is necessary to measure subjective discrimination perception from the perspective of the elderly themselves. However, some studies show that discrimination perception accurately reflects the objective discrimination of the individual (Ruggiero and Taylor, 1995). Higher discrimination perception reduces the happiness of the elderly (Garstka et al., 2004), and it increases their risk of death (Barnes et al., 2008). Therefore, it is necessary to study the discrimination perception of the elderly and its impact on life satisfaction.

Discrimination perception refers to the individual’s perception of inequitable, negative, or harmful treatment. Such unjust treatment can be manifested as an actual action, an attitude of rejection or an unreasonable social system (Major et al., 2002; Pascoe and Richman, 2009; Tom, unpublished). For the elderly, discrimination perception refers to unfair, negative, or injurious treatment of the elderly, which is a subjective reflection of ageism. Most previous research on perceived discrimination have been based on the rejection-identification model to explore the impact on well-being. This model holds that perceived discrimination has a negative impact on well-being, and internal group identification plays a positive mediating role in this relationship (Branscombe et al., 1999). However, in this study, we applied this model to elderly people to explore the relationship between perceived discrimination and life satisfaction for several reasons: (1) life satisfaction is the cognitive component of subjective well-being (Shin and Johnson, 1978), it can be used as a good indicator of well-being; (2) life satisfaction directly reflects the quality of life (Shin and Johnson, 1978), for the elderly, high quality of life may be more important, because it is closely related to health. Therefore, we used life satisfaction instead of well-being in the rejection-identification model. Previous studies of this kind of substitution have also produced similar results with the original model (Gimao et al., 2012; Stronge et al., 2016). However, ecological system theory considers that it is necessary to conduct a comprehensive investigation in combination with different natural environments and specific social backgrounds. This theory divides the individual behavioral system into four levels, from small to large: the micro-system, middle system, external system, and macro-system (Zhu, 2005). According to this theory, research should be conducted at different system levels. However, most previous studies of the elderly have been analyzed at the microsystem level. This is in conformity with the United Nations’ proposal to “build a society for all ages” in 1995. Therefore, research of the elderly advocated in today’s society should also be based on the macro-system. Thus, taking ecological system theory as our framework, we explored the influence of perceived discrimination on life satisfaction in the elderly, from a macro-system and micro-system perspective.

### Analysis of the Macro-System and Micro-System

When studying at the macro-system level, it is necessary to apperceive the internal group and the outgroup as one group, and the internal group identity will change accordingly. The common group identity model suggests that when individuals change the cognitive representation of the two separated groups into one group, they will redefine the inner-group and out-group, and form a common identity (Gaertner and Dovidio, 2012). When the upper group is a “nation,” the common identity is expressed as an individual’s knowledge and acceptance of his or her country membership, that is, national identity. This is a special expression of internal group identity (Yin and Zhang, 2015). There are two reasons for choosing national identity: (1) Examining the role of discrimination in macro-systems, we need to analyze them at the cultural level. On the basis of long-term cultural integration, the Chinese have formed the cultural concept of “pluralistic integration” (a common culture that allows differences to exist, and the commonality is greater than the difference), which promoted the emergence of national identity; (2) although there are many ethnic groups in China, they all share common benefit and common political systems. Therefore, we believe that national identity plays a very important role in Chinese psychology and behavior, leading to hypothesis 1 of our study: that perceived discrimination in elderly people would have a direct negative effect on their life satisfaction, and would have indirect negative effects through national identity.

When examining the role of perceived discrimination at the micro-system level, we need to consider the environment that directly influences the life of the elderly. At present, Chinese society is in a stage of transformation. The community has gradually replaced the status of the family and has become the basic organizational unit of the life of the elderly. Therefore, in this study, we regarded the community as a micro-system for the elderly. However, in community life, the sense of community connects the elderly with other members, playing a very important role in their lives. Sense of community refers to the perception of belonging and dependence, and personal responsibility to individuals and collectives (Dalton et al., 2007). Previous studies have shown that sense of community is an important factor affecting individual life satisfaction. The stronger the sense of community, the higher the satisfaction of people’s lives (Baker and Palmer, 2006; Kutek et al., 2011). In addition, the influence of discrimination perception on the sense of community is considered by relative deprivation theory. Individuals will evaluate their position and situation by comparing themselves to others (Mummendey et al., 1999). When individuals perceive discrimination from members of the group, they experience the deprivation of their basic rights and feel that their influence on the group is gradually declining. At the same time, experience reduces the emotional connection with group members, decreases the sense of belonging and recognition of the whole group, and decreases the sense of community. This suggests that sense of community may also be a mediator of discrimination perception and life satisfaction. Therefore, hypothesis 2 of this study was that the elderly’s perceived discrimination would be negatively related to their life satisfaction through the sense of community.

### The Relationship Between the Macro-System and Micro-System

**FIGURE 1:**
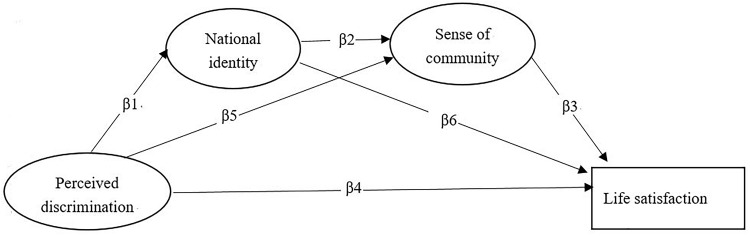
The hypothesis model of the relationship between perceived discrimination and life satisfaction.

With regard to the relationship between the macro-system and micro-system, ecological system theory suggests that there are many connections between them. The ecosystem theory also posits that as time goes on, individual microsystems will change, and this change is likely to be influenced by some aspects of other horizontal systems. Specific to the relationship between national identity and the sense of community, the common internal group identity model holds that an individual’s national identity is an identity common to the upper group, which will enhance the upper group ownership and identity of the individual, and weaken the boundaries of the original and internal groups (Gaertner and Dovidio, 2012). Enhancing the emotional connection among members of the upper group strengthens the sense of community to a certain extent. Therefore, we expect that national identity will also have some influence on the sense of community. Previous studies have also found that individuals’ group identities have a strong predictive effect on the overall sense of community (Obst and White, 2005) Therefore, we hypothesize that perceived discrimination has a direct negative impact on life satisfaction, and indirectly affects life satisfaction through national identity and community sense. The chain mediator model is shown in Figure 1, which includes three intermediary paths: ββ1β6; β5β3; β1β2β3 (Taylor et al., 2008).

## Materials and Methods

### Participants and Procedure

Participants came from two rural communities in Anhui and Shandong Province, respectively, and one urban community in Anhui Province. The participants in the study must be a member of one of the three communities. With the help of community workers, we randomly sent invitations to 500 people over the age of 60 to participate in the study, 55 of whom refused to participate in the survey. After the completion of the questionnaires, we preliminarily sorted it and found that 20 participants did not complete the questionnaire because they quit the survey halfway, and 34 of the participant’s completed questionnaires were invalid. 391 valid questionnaires were finally confirmed (78.20%). Before the survey, we reconfirmed that participants volunteered to participate in the survey and obtained their verbal and written informed consent. After completing all the questionnaires, each participant was given a gift. The demographic data of the participants in the survey were collected. The ages of them ranged from 60 to 101 (more demographic information is presented in Table 1). The experimenter and trained assistants helped participants who were illiterate or had difficulty in completing the questionnaires. The questionnaires took approximately an hour to complete.

**Table 1:** Demographics of the study’s participants.

Demographic variables		*n*	%
Gender	Male	121	30.9
	Female	270	69.1
Community type	Urban community	129	33.0
	Rural community	262	67.0
Education level	Illiteracy	134	34.3
	Primary school	122	31.2
	High school or above	135	34.5
Economic situation (RMB, ¥)	Poor	52	13.3
	Medium	316	80.8
	Rich	23	5.9
Widow or widower	No	219	56.0
	Yes	172	44.0
Health level	Poor	36	9.2
	Medium	208	53.2
	Good	147	37.6

*N = 391.*

### Ethics Statement

This study was carried out following approval by the Ethics Committee of the Psychological Experiment Teaching Center of Nanjing Normal University, with written informed consent obtained from all participants in accordance with the Declaration of Helsinki.

### Measures

#### Perceived Discrimination

The perceived discrimination was measured by the revised version of the Perceived Personal Discrimination Measure (McGarrity and Huebner, 2014). This questionnaire is composed of typical discrimination cases experienced with 16 items, for example, “you have been treated unfairly by service workers (such as: clerk, waiter, bank worker, etc.), because you are elderly,” “you have been pushed or threatened by young people, because you are elderly,” and “You have been called a bad name by other old people.” Each item was scored on a 5-point scale, ranging from 1 (never) to 5 (very much). The participants judged the frequency of these events happening to themselves. The three dimensions of the questionnaire are the discriminations perceived by the elderly from three aspects: society, the elderly, and the young people. To make this questionnaire more suitable for Chinese elderly people, we invited two Ph.D. students majoring in psychology - two professors of psychometrics and two foreign students majoring in psychology whose mother tongue is English, to translate the questionnaire in two directions (Chinese and English), until each item of the two language versions has no difference in meaning. The confirmatory factor analysis showed that the fit indexes of the questionnaire are *χ*^2^/df = 3.30, Tucker-Lewis index (TLI) = 0.93, comparative fit index (CFI) = 0.94, and root mean square error of approximation (RMSEA) = 0.077. The Cronbach’s alpha coefficient for this scale was 0.86. It shows that the scale has good reliability and validity. These indicate that the revised version of Perceived Personal Discrimination Measure has good reliability and validity.

### National Identity

We chose three dimensions of the national identity questionnaire to measure the national identity of the Chinese elderly. The three dimensions were “national heritage,” “cultural homogeneity,” and “consumer ethnocentrism.” This questionnaire was revised by Cui and Adams (2002) and contains 8 items. This questionnaire has been used many times in the United States, France and Russia, showing good reliability and validity (Cui and Adams, 2002). We applied it to Chinese subjects in this study, each item has changed accordingly, for example, “I think the Chinese have cultural attributes that other people do not have,” “I think the Chinese have a common background,” and “Although it may make me spend more time or money, I still like to buy Chinese products.” We have also adopted the same translation process as the Perceived Personal Discrimination Measure to ensure that the understanding of the questionnaire is not different from the original version. Each item was scored on a 5-point scale, ranging from 1 (strongly disagree) to 5 (strongly agree). Confirmatory factor analysis showed that the fit indexes for *χ*^2^/df = 3.20, TLI = 0.90, CFI = 0.94, and RMSEA = 0.076. The Cronbach’s alpha coefficient for the questionnaire was 0.934. These indicate that the revised version of national identity questionnaire has good reliability and validity.

### Sense of Community

The sense of community was measured by using a brief sense of community questionnaire (Long and Perkins, 2003). The questionnaire includes four dimensions: needs satisfaction, membership, influence, and emotional connection, and eight items, for example, “I can get what I need in the community,” and “I feel I have ties with every member of the community.” Each item was scored on a 5-point scale, ranging from 1 (strongly disagree) to 5 (strongly agree). The questionnaire was retested by Peterson et al. (2008), who supported the definition model of the original questionnaire. This questionnaire has been used many times in China, showing good reliability and validity. Confirmatory factor analysis also showed that the fit indexes for *χ*^2^/df = 2.30, TLI = 0.97, CFI = 0.98, and RMSEA = 0.058. The Cronbach alpha coefficient of this scale was 0.935. These indicate that the revised version of the national identity questionnaire has good reliability and validity and is also suitable for the Chinese elderly sample.

### Life Satisfaction

We used the Life Satisfaction Questionnaire to measure life satisfaction. This questionnaire was developed by Diener, and was rechecked by Pavot and Diener (1993), who showed good reliability. The questionnaire contains five items, for example, “In most ways, my life is close to my ideal,” and “The conditions of my life are excellent.” Each item was scored on a 7-point scale ranging from 1 (strongly disagree) to 7 (strongly agree). This questionnaire has been used many times in Chinese elderly (Xie et al., 2014), showing good reliability and validity. The Cronbach alpha coefficient of this study was 0.851.

### Data Analyses

#### Common Method Bias Control

Common method bias is the systematic error caused by using the same method, which will affect the validity of the research results. Generally, there are two ways of controlling common method bias, procedural remedies and statistical remedies. Procedural remedies refer to control the bias in research design and the process of measurement (Yao and Yang, 2017). In this study, we kept strict principles of confidentiality and voluntarism, and asked participants to truthfully fill out each item in the questionnaire. Our measurements are arranged in the senior citizen activity center, where the community provides services specifically for the elderly. The participants can answer freely and authentically in this environment, and after completing the questionnaire, we immediately take back the questionnaire to ensure that no modifications are made. These can partially control the Common method bias. In addition, statistical remedies for common method biases, Harman Single Factor Test is a commonly used method. The results showed that The eigenvalue of eight factors are greater than 1, and The factor with the largest eigenvalue can explained 27.02% of the variance, which is less than the critical standard of 40%. This shows that common method bias in this study is not serious and will not have a significant impact on the study results.

#### Structural Equation Modeling

There are four steps to construct the structural equation modeling in our study: (1) Data feature checking. We need to check whether the data accord with a multivariate normal distribution, and has no serious collinearity problems. If the data do not conform to the normal distribution, we need to transform them accordingly before we can carry out the next statistical analysis. (2) Testing of measurement model. Using confirmatory factor analysis to test whether the fitting index of measurement model conformed to the requirements; (3) Testing of structural equation model. Testing whether the fitted index of the constructed structural equation model conformed to the requirements. And (4) model modification. If the model does not meet the statistical requirements, it can be modified appropriately (Li, 2011).

#### The Multiple Mediator Effect Test

We used the “bootstrap test” to examine the multiple mediation effects. We randomly sampled one person from the participants and repeated this action 1000 times from all the subjects and estimate whether the mediation effect was significant by the significance of the confidence interval (Taylor et al., 2008).

## Results

### Descriptive Statistics

We carried out descriptive analysis and correlation analysis of the data and the results are showed in Table 2. Perceived discrimination was significantly negatively correlated with national identity, sense of community, and life satisfaction. National identity, sense of community, and life satisfaction were significantly positively correlated with each other. This preliminarily showed that the relationship between the four variables of Chinese elderly people.

**Table 2:** Means, SD, and intercorrelations.

		1	2	3	4
1.	Perceived discrimination	1			
2.	National identity	-0.221**	1		
3.	Sense of community	-0.242**	0.591**	1	
4.	Life satisfaction	-0.309**	0.311**	0.392**	1
*M*		26.89	28.82	31.42	22.95
*SD*		9.01	3.66	3.94	5.57

*N = 391. ^∗∗^p < 0.01, all tests were two-tailed.*

The predictive power of the demographic variables on perceived discrimination, national identity, sense of community and life satisfaction are shown in Table 3. The results indicated that the main effects of education level, economic situation and health level on perceived discrimination were significant (*F* = 7.216, *p* < 0.05; *F* = 6.096, *p* < 0.01; *F* = 4.395, *p* < 0.05, respectively), the main effect of gender on national identity was significant (*F* = 5.352, *p* < 0.05), and the main effects of economic situation and health level on life satisfaction were significant (*F* = 16.796, *p* < 0.001; *F* = 8.549, *p* < 0.001, respectively). The main effects of community type and widow or widower on all dependent variables were not significant. Thus, it was inferred that gender, education level, economic situation and health level have significant effects on the construction of the chain mediation model.

**Table 3:** Multivariate analysis of variance of demographic variables.

Demographic variables	Dependent variable	*F*	*η^2^*
Gender	Perceived discrimination	1.783	0.005
	National identity	5.352*	0.014
	Sense of community	0.057	0.000
	Life satisfaction	0.715	0.002
Community type	Perceived discrimination	2.752	0.007
	National identity	1.308	0.003
	Sense of community	3.920	0.010
	Life satisfaction	2.953	0.008
Education level	Perceived discrimination	7.216*	0.036
	National identity	0.115	0.001
	Sense of community	0.212	0.001
	Life satisfaction	0.653	0.003
Economic situation	Perceived discrimination	6.096**	0.031
	National identity	0.389	0.002
	Sense of community	0.283	0.001
	Life satisfaction	16.796***	0.081
Widow or widower	Perceived discrimination	0.890	0.002
	National identity	3.274	0.009
	Sense of community	0.148	0.000
	Life satisfaction	0.133	0.000
Health level	Perceived discrimination	4.395*	0.023
	National identity	0.710	0.004
	Sense of community	2.914	0.015
	Life satisfaction	8.549***	0.043

*^∗^p < 0.05, ^∗∗^p < 0.01, and ^∗∗∗^p < 0.001.*

### Construction of the Chain Mediation Model

**FIGURE 2:**
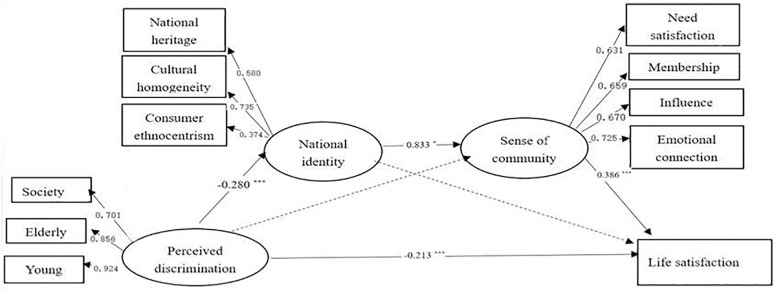
The multiple mediator model of the perceived discrimination and life satisfaction (the dotted line is not significant path). Perceived discrimination, national identity and sense of community are latent variables, and have some different indicators. Each indicator is the sum scores of dimensions of latent variables; ^∗^*p* < 0.05 and ^∗∗∗^*p* < 0.001.

We tested the skew and kurtosis of the data to assess whether the data conform to the normal distribution. The results show that the absolute value of skew (Zs) and kurtosis (Zk) was less than 1.96, which indicate that the data in this study conform to the first step in constructing structural equation. Next, we evaluate the collinearity of the data. The results show that the tolerance (0.932, 0.644, and 0.637) to be greater than 0.10, and the variance inflation factor (1.073, 1.553, and 1.570) was less than 10. All of these indicate that the data in this study do not have serious collinearity problems. The analysis of the measurement model showed that the measurement model fit the data well (RMSEA = 0.071, CFI = 0.953, TLI = 0.933, and SRMR = 0.043). We also assessed the theoretical model and found that the data fitting index is very good (RMSEA = 0.067, CFI = 0.953, TLI = 0.933, and SRMR = 0.045) (see Figure 2).

### The Mediating Effect Test

We used the “bootstrap test,” repeated sampling 1,000 times, and calculated 95% confidence intervals (CI) to assess whether our hypothetical mediation models were significant. The results show that the path from perceived discrimination to life satisfaction through the chain of national identity and sense of community was significant. Confidence intervals were 95% CI [-0.233, -0.020] and did not contain 0, indicating that the chain mediating effect was significant; the size of the effect was 31.53%. However, the other paths from perceived discrimination to life satisfaction through national identity or sense of community were non-significant. Confidence intervals were 95% CI [-0.102, 0.038] and [-0.120, 0.152]. The total mediating effect value was -0.101, *p* < 0.05, and the 95% CI [-0.181, -0.035] did not contain 0. This indicating that the total mediating effect was significant; the size of the total mediating effect was 32.17%.

## Discussion

The present study investigated the influence of perceived discrimination on life satisfaction at two systems levels of the Chinese elderly, and we selected national identity and sense of community as mediating variables to investigate this effect. The results suggest that perceived discrimination has a direct negative impact on life satisfaction of the elderly, and indirectly through identity and community sense. National identity and sense of community as a chain structure mediate the relationship the relationship between perceived discrimination and life satisfaction. The size of the total mediating effect was 32.17%. This indicates that the constructed hypothetical model has a certain explanatory power to describe the negative influence of the elderly in China perceived discrimination. The model featured one significant path (see Figure 1), which was from perceived discrimination to life satisfaction through the chain of national identity and sense of community (β1–β2–β3). However, the special mediating effects of national identity (β1–β6) and sense of community (β5–β3) were not significant, signifying that national identity and sense of community must be linked together to play a strong mediating role, and meaning that the macro-system and micro-system can be very effective if they can be combined together. This also confirms the viewpoint of ecosystem theory, which believes that the individual is not living in a single social environment, but that the micro-systems (e.g., family, friends, and community) and macro-systems (e.g., nation, society, and so on) of individuals’ behavior will have a certain impact on their development. In addition, the present study found that the perceived discrimination negatively predicts the national identity of Chinese elderly people. This result shows some differences from previous studies, such as Garstka et al. (2004) who found that the elderly perceived age discrimination positively predicted the age identity, which then positively predicted the psychological well-being. The possible reasons are as follows: firstly, in previous studies of perceived discrimination, the object group’s boundary was very distinct and the status is difficult to transform, such as immigrants, and black people in a certain area. After being discriminated against, the same experience will prompt them to form a strong internal group identification and alleviate the negative effects of discrimination. The elderly is different from other groups, as they have no clear group boundary and a certain fluidity, particularly with the “Chinese dream” strategy put forward (this strategy was a call to realize the great rejuvenation of the Chinese nation), where all Chinese people are forming a community of common destiny. The old people are building a wider national identity, within the boundary of all Chinese people. Therefore, discrimination against the elderly comes from within the group, it cannot enhance the effect of national identity. Secondly, under the influence of the concept of Chinese collectivism, the behavior of the individual represents the collective or the society in which he or she is located. When the elderly perceive discrimination, what first comes to mind is the injustice from the whole group, i.e., their collective or the whole society. Therefore, the elderly’s identification with the whole collective or society will also decrease correspondingly. Thirdly, the participants in previous studies perceived the discrimination that came from external groups, while the elderly in this study perceived discrimination that come from within the group, resulting in different results.

### Implications for Researchers and Practitioners

The present study has some implications for researchers and community workers. This study reveals that factors from various systems can play an important role in the relationship between perceived discrimination and life satisfaction. Not only the micro-system and macro-system but also factors from the middle system (e.g., family status-peer relationship) and the external system (e.g., working environment of parents) may be important. The common role of all systems remains to be explored. For community workers, they can enhance the elderly’s sense of community and reduce the negative impact of perceived discrimination on them, so as to enhance the elderly life satisfaction. Therefore, it is very important for community workers to strengthen the connection and sense of belonging among community members, especially the elderly.

### Limitations

This study also has some limitations. First, although this research is based on ecosystem theory, it only explored the macro-system and micro-system, and lacked discussion of the middle system and external system. Follow-up research should pay more attention to these systems. Second, the results may be affected by some factors, which may lead to errors, such as social desirability effect of the participants.

In conclusion, the present study provides some insight into the relationship between perceived discrimination and life satisfaction. Specifically, we found that perceived discrimination negatively influenced life satisfaction directly, and indirectly through national identity and sense of community in the Chinese elderly. The relationship between perceived discrimination and life satisfaction was partially mediated by the chain of national identity and sense of community. From the analysis on the mediating effects, we can conclude that national identity and community sense as a chain structure play an important role in the relationship between perceived discrimination and life satisfaction of the Chinese elderly.

## Author Contributions

JY participated in the design, data collection, data analysis, data interpretation, and drafting of the early version of the article. LY participated in the drafting and revising of the article. XH participated in the design and drafting of an early version of the article. YL participated in questionnaire development.

## Conflict of Interest Statement

The authors declare that the research was conducted in the absence of any commercial or financial relationships that could be construed as a potential conflict of interest.

## References

[B1] Baker D. A. Palmer R. J. (2006). Examining the effects of perceptions of community and recreation participation on quality of life. *Soc. Indicat. Res.* 753 395–418. 10.1007/s11205-004-5298-1

[B2] Barnes L. L. de Leon C. F. Lewis T. T. Bienias J. L. Wilson R. S. Evans D. A. (2008). Perceived discrimination and mortality in a population-based study of older adults. *Am. J. Public Health* 98 1241–1247. 10.2105/AJPH.2007.114397 18511732 PMC2424090

[B3] Bowling A. Browne P. D. (1990). Association with life satisfaction among very elderly in People Living in a deprived part of inner London. *Soc. Sci. Med.* 31 1003–1009. 10.1016/0277-9536(90)90112-6 2255958

[B4] Branscombe N. R. Schmitt M. T. Harvey R. D. (1999). Perceiving pervasive discrimination among African Americans: implications for group identification and well-being. *J. Pers. Soc. Psychol.* 77 135–149. 10.1037/0022-3514.77.1.135

[B5] Chen S. Yue G. (2001). Research of urban residents’ life satisfaction and its influencing factors. *J. Psychol. Sci.* 24 664–765. 10.16719/j.cnki.1671-6981.2001.06.007

[B6] Cui C. C. Adams E. I. (2002). National identity and natid: an assessment in yemen. *Int. Mark. Rev.* 19 637–662. 10.1108/02651330210451953

[B7] Dalton J. H. Elias M. J. Wandersman A. (2007). *Community Psychology Linking Individuals and Communities.* Connecticut: Cengage Learning.

[B8] Gaertner S. L. Dovidio J. F. (2012). “Reducing intergroup bias: the common intergroup identity model,” in *Handbook of Theories of Social Psychology*, eds Lange P. A. M. V. Kruglanski A. W. Higgins E. T. (Thousand Oaks, CA: Sage), 439–457.

[B9] Garstka T. A. Schmitt M. T. Branscombe N. R. Hummert M. L. (2004). How young and older adults differ in their responses to perceived age discrimination. *Psychol. Aging* 19 326–335. 10.1037/0882-7974.19.2.326 15222826

[B10] Gimao L. S. Schmitt M. T. Outten H. R. (2012). Perceived Discrimination, group identification, and life satisfaction among multiracial people: a test of the rejection-identification model. *Cult. Divers. Ethnic. Minor. Psychol.* 18 319–328. 10.1037/a0029729 23066642

[B11] Gow A. J. Pattie A. Whiteman M. C. Whalley L. J. Deary I. J. (2007). Social support and successful aging: investigating the relationships between lifetime cognitive change and life satisfaction. *J. Individ. Differ.* 28 103–115. 10.1027/1614-0001.28.3.103

[B12] Heaven P. (1989). Extraversion, Neuroticism and Satisfaction with Life among Adolescents. *Pers. Individ. Differ.* 10 489–492. 10.1016/0191-8869(89)90029-9

[B13] Huebner E. S. (1991). Correlates of life satisfaction in children. *School Psychol. Q.* 6 103–111. 10.1037/h0088805

[B14] Iversen T. N. Larsen L. Solem P. E. (2009). A conceptual analysis of ageism. *Nordic Psychol.* 61 4–22. 10.1027/1901-2276.61.3.4

[B15] Kahn J. H. Hessling R. M. Russell D. W. (2003). Social support, health, and well-being among the elderly: what is the role of negative affectivity? *Pers. Individ. Differ.* 35 5–17. 10.1016/S0191-8869(02)00135-6

[B16] Kutek S. M. Turnbull D. Fairweather-Schmidt A. K. (2011). Rural men’s subjective well-being and the role of social support and sense of community: evidence for the potential benefit of enhancing informal networks. *Aust. J. Rural Health* 19 20–26. 10.1111/j.1440-1584.2010.01172.x 21265921

[B17] Li S. D. (2011). Testing mediation using multiple regression and structural equation model ing analyses in secondary data. *Eval. Rev.* 35 240–268. 10.1177/0193841X11412069 21917711

[B18] Long D. A. Perkins D. D. (2003). Confirmatory factor analysis of the sense of community index and development of a brief SCI. *J. Commun. Psychol.* 31 279–294. 10.1002/jcop.10046

[B19] Major B. Quinton W. McCoy S. (2002). Antecedents and consequences of attributions to discrimination: theoretical and empirical advances. *Adv. Ex. Soc. Psychol.* 34 251–329. 10.1016/S0065-2601(02)80007-7

[B20] McGarrity L. A. Huebner D. M. (2014). Is being out about sexual orientation uniformly healthy? The moderating role of socioeconomic status in a prospective study of gay and bisexual men. *Ann. Behav. Med.* 47 28–38. 10.1007/s12160-013-9575-6 24307473

[B21] Mellor D. Stokes M. Firth L. Hayashi Y. Cummins R. (2008). Need for belonging, relationship satisfaction, loneliness, and life satisfaction. *Pers. Individ. Differ.* 45 213–218. 10.1016/j.paid.2008.03.020

[B22] Mummendey A. Kessler T. Klink A. Mielke R. (1999). Strategies to cope with negative social identity: predictions by social identity theory and relative deprivation theory. *J. Pers. Soc. Psychol.* 76 229–245. 10.1037/0022-3514.76.2.229 10074707

[B23] Obst P. L. White K. M. (2005). An exploration of the interplay between psychological sense of community, social identification and salience. *J. Community Appl. Soc. Psychol.* 15 127–135. 10.1002/casp.813

[B24] Pascoe E. A. Richman L. S. (2009). Perceived discrimination and health: a meta-analytic review. *Psychol. Bull.* 135 531–554. 10.1037/a0016059 19586161 PMC2747726

[B25] Pavot W. Diener E. D. (1993). Review of the satisfaction with life scale. *Psychol. Assess.* 5 164–172. 10.1037/1040-3590.5.2.164

[B26] Peterson N. A. Speer P. McMillan D. (2008). Validation of a brief sense of community scale: confirmation of the principal theory of sense of community. *J. Commun. Psychol.* 36 61–73. 10.1002/jcop.20217

[B27] Ruggiero K. M. Taylor D. M. (1995). Coping with discrimination: how disadvantaged group members perceive the discrimination that confronts them. *J. Pers. Soc. Psychol.* 68 826–838. 10.1037/0022-3514.68.5.826 9107006

[B28] Shin D. C. Johnson D. M. (1978). Avowed happiness as an overall assessment of the quality of life. *Soc. Indicat. Res.* 5 475–492. 10.1007/BF00352944

[B29] Stronge S. Sengupta N. K. Barlow F. K. (2016). Perceived discrimination predicts increased support for political rights and life satisfaction mediated by ethnic identity: a longitudinal analysis. *Cult. Divers. Ethnic. Minor. Psychol.* 22 359–368. 10.1037/cdp0000074 26460667

[B30] Taylor A. B. MacKinnon D. P. Tein J. Y. (2008). Tests of the three-path mediated effect. *Organ. Res. Methods* 11 241–269. 10.1177/1094128107300344 25316269

[B31] Xie X. Duan H. Gu C. (2014). Older adults’ attachment affects their satisfaction with life: the mediating effect of loneliness. *J. Psychol. Sci.* 37 1421–1425. 10.16719/j.cnki.1671-6981.2014.06.023

[B32] Yao J. Yang L. (2017). perceived prejudice and the mental health of chinese ethnic minority college students: the chain mediating effect of ethnic identity and hope. *Front. Psychol.* 8:1167 10.3389/fpsyg.2017.01167 28744249 PMC5504151

[B33] Yin R. Zhang F. (2015). The Mechanism of Group Identity in Collective Action. *Adv. Psychol. Sci.* 23 1637–1646. 10.3724/SP.J.1042.2015.01637

[B34] Zhai Z. Chen J. Li L. (2017). Future trends of china’s population and aging: 2015-2100. *Popul.Res.* 41 60–71.

[B35] Zhu B. (2005). The reliabilities and validities of the Seven-factor Chinese Personality Sca le. *J. Psychol. Sci.* 28 922–925. 10.16719/j.cnki.1671-6981.2005.04.038 19239534

